# Lab-on-a-molecule and multi-analyte sensing

**DOI:** 10.3389/fchem.2024.1393308

**Published:** 2024-04-18

**Authors:** David C. Magri

**Affiliations:** Laboratory of Molecular Logic Gates, Department of Chemistry, Faculty of Science, University of Malta, Msida, Malta

**Keywords:** lab-on-a-molecule, molecular logic gate, multi-analyte detection, chemosensor, fluorescence sensing

## Abstract

The concept of a lab-on-a-molecule, which was proposed just short of two decades ago, has captured the imagination of scientists. From originally being proposed as an AND logic gate driven by three chemical inputs as a direct way of detecting congregations of chemical species, the definition of what constitutes a lab-on-a-molecule has broadened over the years. In this review, molecules that can detect multiple analytes by fluorescence, among other techniques, are reviewed and discussed, in the context of molecular logic and multi-analyte sensing. The review highlights challenges and suggestions for moving the frontiers of research in this field to the next dimension.

## 1 Introduction

The first purposely designed lab-on-a-molecule was a chemical entity built with my own hands (Magri et al., 2006a). At the time, I was a post-doctoral research fellow at Queen’s University Belfast in Northern Ireland working in the laboratory and office of Prof. A. Prasanna de Silva. The goal of the project was to synthesize and demonstrate an AND logic gate driven by three chemical inputs, with an optical output, as a direct way of detecting a congregation of chemical species in water.

Having accomplished all of this, the next logical task was to prepare a manuscript. During the preparation of the manuscript for submission to the *Journal of the American Chemical Society*, a considerable amount of thought and importance was placed on the first lines of the manuscript. How were we going to convince the editor and reviewers of the importance of detecting for three things, rather than just two? Well, after much thought, we reasoned that a third dimension in geometry is a big deal and changes our reality from the concept of area to volume (Abbott, 1950). Then, there is the revolutionary transistor, which, with a third electrode, can regulate current or voltage flow in addition to performing signal amplification; the latter is something a two-electrode one-way diode cannot do (Millman and Grabel, 1988).

At the time, there was much fanfare regarding the state-of-the-art blood analyte Opti Medical cassette and portable device, which provides a readout for six blood serum analytes (Tusa and He, 2005; Yao and de Silva, 2023). The vision put forward in 2006 stems within the context of molecular logic-based computing (de Silva and Uchiyama, 2007; Andréasson and Pischel, 2010; Szaciłowski, 2012; de Silva, 2013; Ling et al., 2015; Andréasson and Pischel, 2018; Erbas-Cakmak et al., 2018; Yao et al., 2020; Magri, 2021). Rather than testing for many disease parameters by many separate tests, followed by manual consideration of the data by a practitioner, what if a medical condition could be directly diagnosed by a single rapid test. Such a molecular device would be a lab-on-a-molecule since a clinically relevant result would emerge from on-board information processing of several sensory channels simultaneously (Magri et al., 2006b). In other words, a lab-on-a-molecule could test for any number of disease markers and provide a diagnostic “Yes” or “No” decision on the condition of the patient.

In 2015, an influential review article on the “Design strategies for lab-on-a-molecule probes and orthogonal sensing” was published in *Chem. Soc. Rev*. (Chen et al., 2015). It has blurred the original vision of what constitutes a lab-on-a-molecule. In addition to this article, at least two other prior research articles have redefined a lab-on-a-molecule as a chemical entity that can quantitatively sense *two* or more analytes in a multi-analyte mixture. A *Web of Science* survey of the primary literature revealed there are 21 publications with lab-on-a-molecule in their titles. When the survey is extended to a topic search (excluding reviews) the number of publications is 27. However, of these lists of publications, perhaps in only one-quarter of the studies, it has been demonstrated that a molecule detects for three chemical species. In this review, only molecules that detect for a minimum of three chemical species are presented.

## 2 Lab-on-a-molecule and multi-sensing chemosensors

Within lab-on-a-molecule **1**, three receptors are present, each specific for an individual analyte. A benzo-15-crown-5 ether binds Na^+^, a tertiary amine binds H^+^, and a phenyliminodiacetate binds Zn^2+^. These components were selected, to some extent, for synthetic convenience. The fluorescent reporter is the planar blue-emitting anthracene fluorophore. These four components are covalently attached and separated by methylene spacers to minimize the distance for photoinduced electron transfer (PET) between the receptors and the excited fluorophore (de Silva et al., 2009). The sensing approach represented by **1** entailed an engineered molecule with a receptor site for each specific analyte examined within the context of molecular logic (Szaciłowski, 2012; de Silva, 2013). In water with high concentration levels of Na^+^, H^+^, and Zn^2+^, the molecule emits blue light with a fluorescent quantum yield (Φ_f_) of 0.02 and a threefold enhancement. The absence of just one of the three analytes prevents the molecule from emitting the blue emission due to PET.


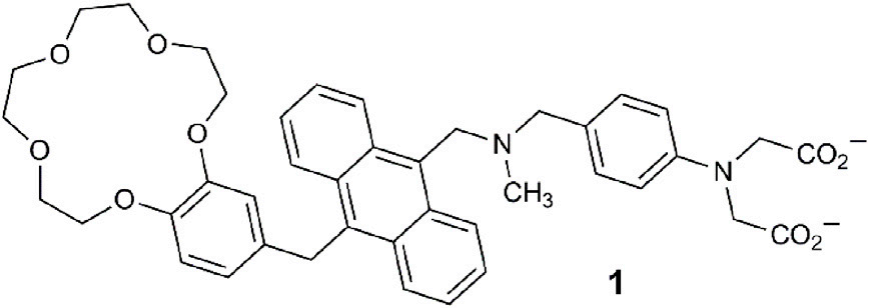


Another paradigm for multi-analyte sensing emerged based on the application of distinguishing heavy metal ions by three techniques (Jiménez et al., 2004). As triple-channel sensing molecules **2** and **3** examine metal ions by cyclic voltammetry and UV-vis spectrometry, in addition to fluorescence. These systems are examples with twisted intramolecular charge transfer (TICT) excited states due to twisting about the anilinic C-N or aromatic vinyl bonds (Rettig, 2005). The output of the chemosensors can, in principle, result from any, some, or all these three methods. A blue shift occurred in the absorbance spectrum of **2** with Pb^2+^, while for **3**, a hypsochromic shift was accompanied by a color change from red to yellow in the presence of Hg^2+^. The emission was quenched by Cu^2+^ and Fe^3+^ by PET or energy transfer. Anodic shifts in the cyclic voltammograms for Hg^2+^, Pb^2+^, and Fe^3+^ result from cationic destabilization of the radical cation. This study nicely illustrated how a single molecule could selectively detect three different ions via three separate outputs. In a review of this work (Callan et al., 2005), it was concluded that “[they were] not considered along with logic systems, since the targets [were] not presented as sets to the molecular device.”


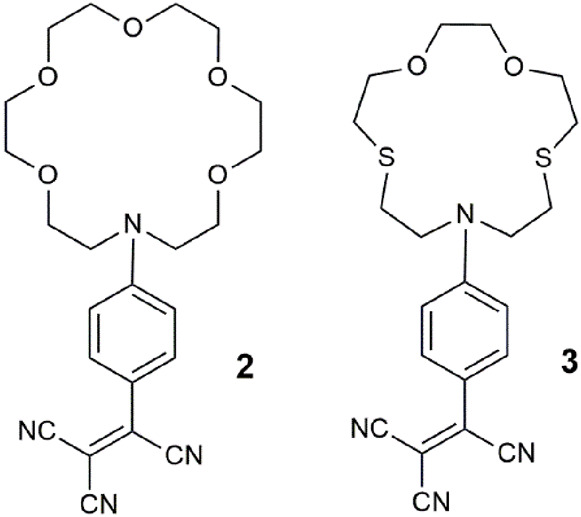


Chemosensor **4** consists of a ruthenium-phenanthroline lumophore and two aza-crown ethers (Schmittel and Lin, 2007). As a quadruple-channel sensing molecule, it exploits the unprecedented application of an array of analytical techniques to detect Cu^2+^ by UV–vis absorbance, Pb^2+^ by luminescence, Pb^2+^ by cyclic voltammetry, and Hg^2+^ by electrogenerated chemiluminescence. The two crown ethers work cooperatively as a single pocket site (Magri et al., 2006a) to bind non-specifically to larger metal ions as demonstrated with Pb^2+^, Hg^2+^, and Cu^2+^ in acetonitrile. A quiet criticism remarked by a colleague at the Molecular Sensors and Molecular Logic Gate conference, shortly after its publication was whether **4** is more akin to a “molecule-in-a-lab” rather than a “lab-in-a-molecule.” A practical challenge of this strategy from the viewpoint of point-of-care application is the miniaturization of the instrumentation into a single compact device. Another example fitting this genre is an iridium (III) complex as a triple-channel chemosensor for cysteine, homocysteine, and tryptophan (Chen and Schmittel, 2013).


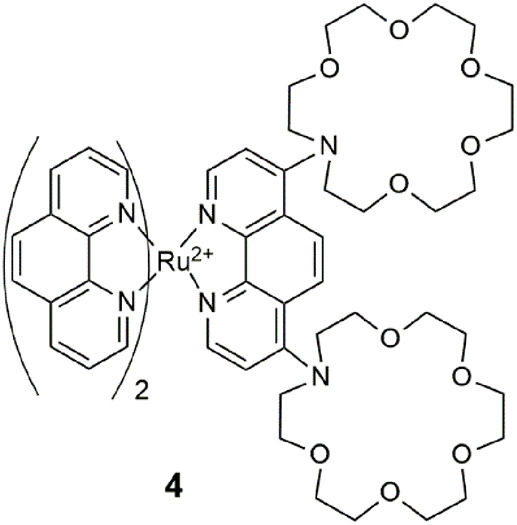


BODIPY-based **5** (Bozdemir et al., 2010) detects for a congregation of three metal ions in a manner similar to **1**, where each receptor is selective for a specific analyte. The benzo-aza-crown, benzo-dithio-aza-crown, and dipicolylamine receptors bind Ca^2+^, Hg^2+^, and Zn^2+^, respectively. The three-input logic gate emits at 656 nm with a bright yellow glow, a fluorescence quantum yield of 0.266 and a threefold enhancement in acetonitrile. The quenching mechanism via the benzo-aza-crown is attributed to PET, while the other two receptors quench the emission by an intramolecular charge transfer (ICT) mechanism.


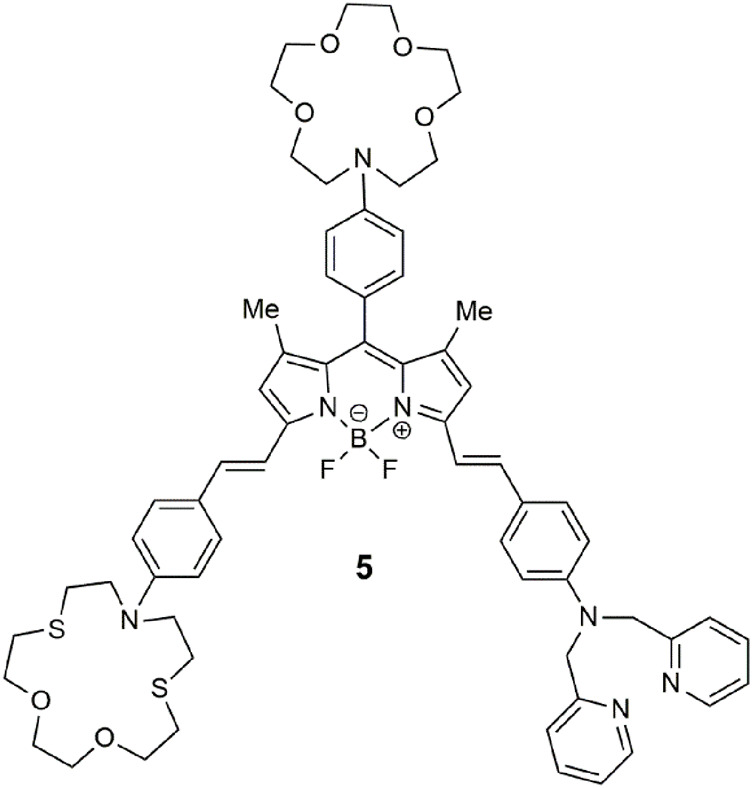


Combinatorial differential fluorescence sensing was proposed for the detection and discrimination of medicines (Rout et al., 2014). The tailor-made molecule **6** has multiple boronic acid receptors and four fluorophores (naphthalene, anthracene, fluorene, and dansyl) and a central amino-*L*-proline (Rout et al., 2012). Binding of various medications results in unique fluorescent signatures due to competing PET, ICT, and Föster resonance energy transfer (FRET) processes. Principle component analysis (PCA) was then applied to the various classes of medication (Wright and Anslyn, 2006). The concept has been extended to other permutations of receptors and fluorophores for chemical inputs that include solvents, metal ions, anions, saccharides, pH, and polarity. Encryption of secret messages (Sarkar et al., 2016) and molecular scale keypad locks (Rout et al., 2013) have further been demonstrated using this approach.


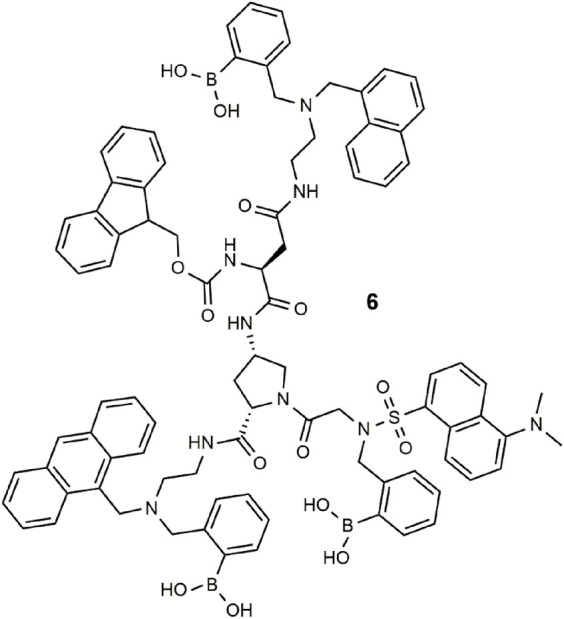


Lab-on-a-molecule **7** (Magri et al., 2014) is an amalgamation of fluorescent Na^+^, H^+^-driven (de Silva et al., 1997a), and Fe^3+^, H^+^-driven AND logic gates (Farrugia and Magri, 2013). It uniquely incorporates three types of chemical equilibria: complexation, acid–base dissociation, and redox equilibria. The benzo-15-crown-5 ether binds Na^+^ and the tertiary amine binds H^+^, while the ferrocene is oxidized by Fe^3+^ to yield ferrocenium and Fe^2+^. PET from the crown receptor, or the alkyl amine, or ferrocene to the anthracene fluorophore competes with fluorescence to quench the emission. The three-input AND logic gate emits an enhanced fluorescence in the presence of high Na^+^, H^+^, and Fe^3+^ levels with a Φ_f_ of 0.072 in methanol. High levels of Na^+^, H^+^, and Fe^3+^ are associated with various cancer cells, which tend to grow quicker than healthy cells, and therefore accumulate higher levels of various analytes. The same trio of analytes are also early warning markers for corrosion of steel and other ferrous metals (Scerri et al., 2021), particularly for automobiles in colder geographic regions where NaCl is used for deicing snowy roadways.


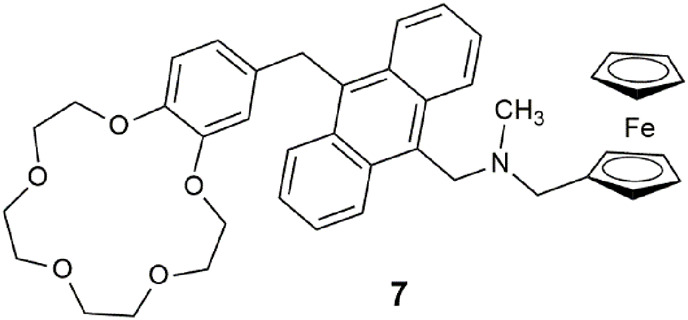


The ferrocene–naphthalimide–boronic acid conjugate **8** is a type of three-input combinatorial INHIBIT logic gate (Li et al., 2015). A fluorescence output is observed with high Fe^3+^, low sodium *L*-ascorbate, and low F^−^. Oxidization of the ferrocene moiety with one equivalent of Fe^3+^ acts as an oxidant to oxidize ferrocene to its radical cation, resulting in a 100-fold fluorescent enhancement at 512 nm in THF. The role of *L*-absorbate is to reduce Fe^3+^ to Fe^2+^ rather than interact with **8** directly. The relationship between these two analytes shows reversible off–on–off switching. F^−^ selectively forms a covalent bond with the boronic ester to form a tetrahedral boronate anion, but it too interferes with Fe^3+^. Hence, Fe^3+^ is the only enabling input, while *L*-ascorbate and F^−^ both disable the output emission by interacting with Fe^3+^. This logic gate is unconventional compared to **1** as it incorporates the operations of redox chemistry and a chemodosimeter (Du et al., 2012).


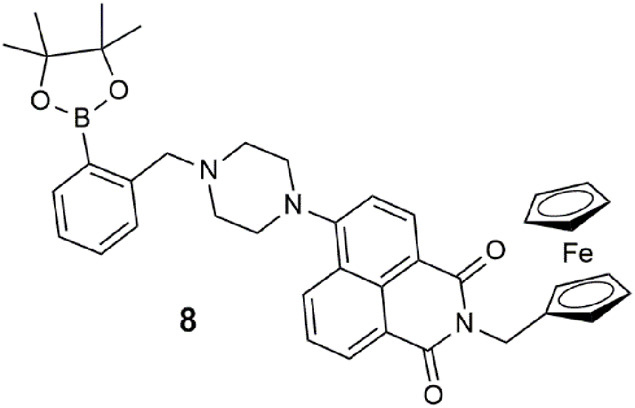


Prototype **9** has some resemblance to **7**, except that the fluorophore is replaced with an 4-amino-1,8-naphthalimide and the arrangement of the module units is different. The input chemicals remain Na^+^, H^+^, and Fe^3+^. A three-input AND logic gate is the predicted functionality based on the PET design concept (Magri, 2021). In practice, the logic gate glowed green under two scenarios in 1:1 MeOH/water: when all three analyte levels were high and when only the H^+^ and Fe^3+^ were present. These two scenarios in isolation are three-input AND (Magri et al., 2006b) and three-input INHIBIT (de Silva et al., 1999) logic operations. With **9**, we have an example of wireless integration of both functions within a three-input AND-INHIBIT-OR circuit. A possible explanation for the visualization of light in the INHIBIT case with only H^+^ and Fe^3+^ could be that Fe^3+^ or, more probably, the Fe^2+^ generated by oxidation of the ferrocene interacts with the benzo-crown ether. This example opens up a different application from a clinical diagnostic viewpoint. Three-input AND logic coincides with a state when all three analytes exceed their respective threshold levels. However, for conditions when one of the three analytes are detrimental to the patient—three-input INHIBIT logic—a scenario results where the desired outcome is a situation when two analytes exceed, and the third is below the threshold levels. A combinatorial two-input INHIBIT-AND logic gate was proposed as a method for intelligent medical screening for a protein or for both DNA and a protein in a biological sample autonomously (Konry and Walt, 2009).


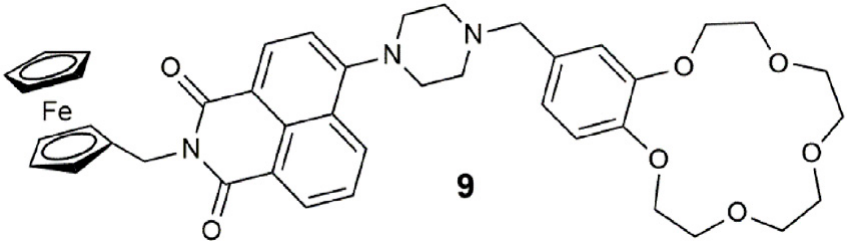


Building on the lessons learnt from **9**, molecule **10** is a bright green-emitting lab-on-a-molecule (Scerri et al., 2019). The previously used benzo-15-crown-5 ether sodium receptor was upgraded to the stronger binding *N*-(2-methoxyphenyl)aza-15-crown-5 ether used in binding Na^+^ in blood serum (Tusa and He, 2005). The aliphatic nitrogen atom within the piperazine moiety binds H^+^. The redox-active ferrocene group for sensing Fe^3+^ remains in the same location. Extra effort was required to synthesize this superior aza-crown ether, but it was worth it. In 1:1 (v/v) MeOH/H_2_O, the emission digitally switches “on” with a 25-fold enhancement and a Φ_f_ of 0.203. The reader is reminded of the significance of this trio of analytes in corrosion detection (Scerri et al., 2021). The related two-input AND logic gates simultaneously detect high levels of Fe^3+^ and H^+^ levels under conditions of excess Na^+^ to accelerate the rate of corrosion of mild steel (Scerri et al., 2021).


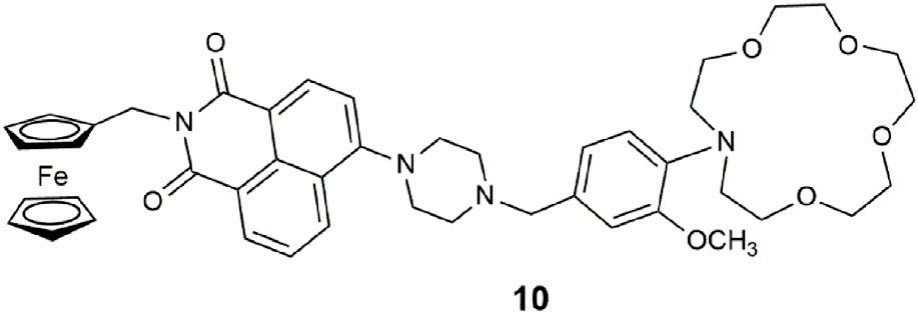


A type of neuron in the forebrain has high levels of glutamate and zinc that are co-released into the synaptic cleft between neurons. Logic gate **11**, based on a hydroxycoumarin-3-aldehyde scaffold, was developed as a prototype for monitoring the co-release of glutamate and zinc from secretory vesicles (Hettie et al., 2014). It was tested in HEPES buffer at an ionic strength of 120 mM NaCl with 1% DMSO with glutamate, Zn^2+^, and OH^−^ (at neutral pH). Rather than relying on intermolecular interactions, the sensing strategy is dependent on the reactivity of the aldehyde functionality where reversible binding of glutamate occurs to form an imine bond. Consequently, a multi-dentate binding pocket forms, whereby Zn^2+^ can bind with the lactone carbonyl oxygen of the coumarin (Bourson et al., 1993) and the N and O atoms of the glutamate. The proton site is the phenolate. An 11-fold fluorescence enhancement is observed under exocytosis conditions with glutamate, Zn^2+^, and neutral pH. This example of a three-input AND logic gate differs from those previously highlighted, in that the receptors are not orthogonal to one another. The molecule functions first as a chemodosimeter to bind the amino acid, which then provides the pocket for Zn^2+^ binding.


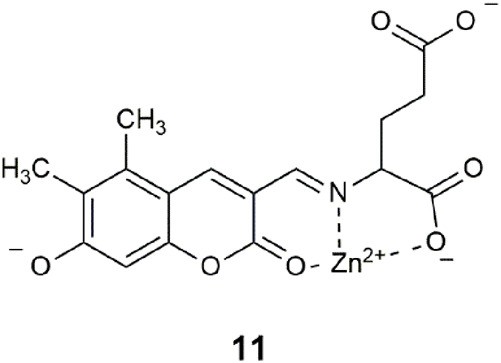


Proteins have rarely features as inputs in multi-analyte logic gates (Wu et al., 2011; McLaughlin et al., 2018). Here, we highlight a logic gate **12** driven by two atomic inputs, H^+^ and Na^+^, and a hydrolase enzyme, as the third input (Wright et al., 2020). *Candida antarctica* lipase B was selected as the biomolecular input after the fluorescence screening of 38 enzymes. The fluorescence response to the three inputs was measured in 1:1 water/DMSO after 30 min at pH 7 with 1.0 M Na^+^. The hydrolysis of the ester by the lipase to the carboxylate anion causes an upward shift in the amine p*K*_a_ value due to the stabilization gained from the electrostatic attraction between the protonated amine and the negatively charged carboxylate. This is a clever way of exploiting an electric field effect (Spiteri et al., 2018). The switching enhancement from ester hydrolysis is a non-optimal factor of 2, but as a proof-of-principle, it meets the minimum output threshold.


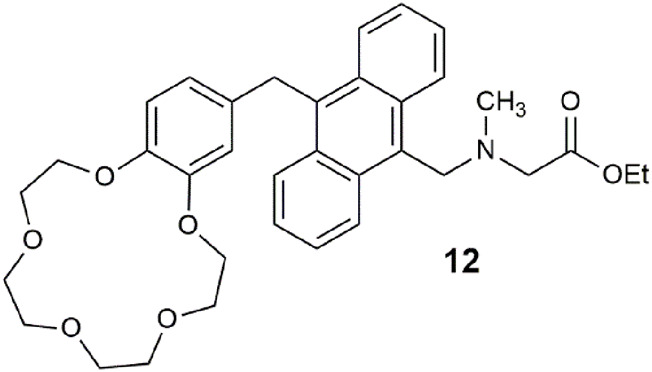


The 3-pyrazolinyl-naphthalimide hybrid **13** operates as a wavelength-reconfigurable dual-output logic gate (Magri and Camilleri, 2023). The benzo-15-crown-5 receptor contributes as the receptor and PET donor. Irradiated with a 365-nm UV lamp, red emission is initially observed (Φ_f_ = 0.095). The capture of Na^+^ reveals orange emission (Φ_f_ = 0.27). When substituted with Mg^2+^, remarkably, the result is a Φ_f_ = 0.40 and white light emission (WLE). The presence of high H^+^ deactivates the excited state by protonation of the pyrazoline atom via charge transfer. The molecular device can function as two INHIBIT gates in parallel with H^+^ as the disabling input. Alternatively, on excitation at 470 nm, **13** is reconfigurable as a three-input OR-INHIBIT combinatorial logic gate. Other related prototypes are envisioned by replacement of the phenyl ring at the naphthalimide with ferrocene, for example, or the introduction of an enzyme input as is with **9** (Wright et al., 2020), or by attachment of the molecular logic gate to a solid support for sensing in water (Vella Refalo et al., 2018, 2019). Other notable rare examples of WLE logic gates are a coumarin–rhodamine coordinated complex with Fe_3_O_4_ nanoparticles for selective detection of ClO^−^ and SCN^−^ ions (Zhi et al., 2015) and Eu^3+^- and Tb^3+^-doped tribochromism coordination polymers (Yang et al., 2017).


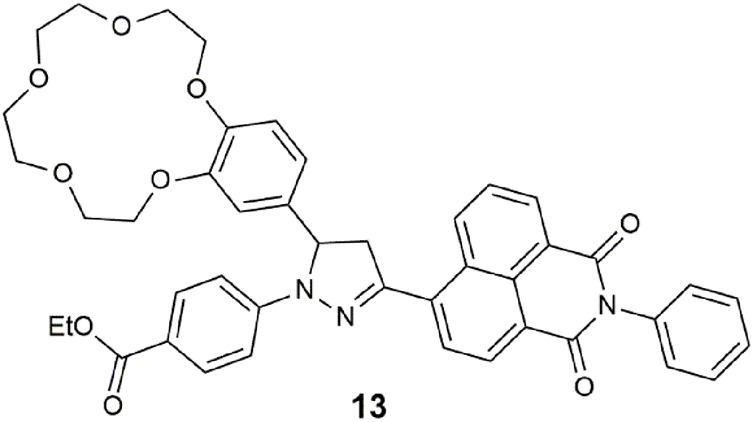


A triple-channel fluorescent probe **14** for the detection and discrimination of cyanide, hydrazine, and hypochlorite was demonstrated in water (Suna et al., 2023). The molecule is engineered on a D-π-A platform with triphenylamine as the electron-donating group, a fluorobenzene-substituted thienothiophene as the π spacer, and a thiobarbituric acid as the electron-accepting group. Excitation with 360 nm light reveals white emission with CN^−^, green emission with hydrazine, and orange emission with ClO^−^. The trio of analytes react with **14** by nucleophilic addition at the vinylthiophene position. The practical uses of the probe were verified with spring water and wastewater and, for hypochlorite, with fruits and vegetables.


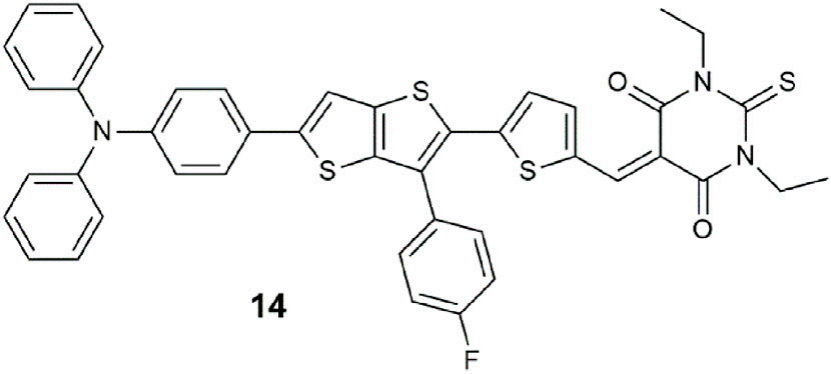


We recently demonstrated that the *cinchona* alkaloids are examples of INHIBIT and combinatorial OR-INHIBIT logic gates in water (Agius and Magri, 2023). Among the examined alkaloids was quinine **15**, the two century old, anti-malarial, and gold standard for measuring fluorescence quantum yields (Φ_f_ = 0.55). The four *cinchona* alkaloids are all commercially available, so there was no need for synthetic effort, unlike the situation for the first INHIBIT logic gate (Gunnlaugsson et al., 2000) and many other INHIBIT logic gates reviewed (Souto and Dias, 2023). Remarkably, the design concepts of PET and ICT are intrinsically found within the framework of **15**. Protonation of the azabicyclo shuts off a PET pathway, while a second protonation at the quinoline nitrogen atom induces a large fluorescence enhancement. The second input is Cl^−^, Br^−^, or I^−^, which disables the emission by collisional quenching. This approach is not the modern ideal for detecting an analyte, yet it remains an applied method used in hospitals for blood diagnostics (Tusa and He, 2005) and for tears via contact lenses (Badugu et al., 2020).

We have also reported a polymeric H^+^, Cl^−^ driven INHIBIT gate **16** based on quinidine by exploiting the vinyl moiety as a linker with acrylamide (Agius and Magri, 2021). The copolymer conserved the fluorescence properties of the quinidine monomer with a 185-fold enhancement and Φ_f_ = 0.56 in water. Future lab-on-a-polymer (Ueda et al., 2007) systems can be envisioned by incorporating additional monomer receptor units. We are optimistic that further screening of fluorescent natural products from a molecular logic viewpoint should reveal other naturally occurring fluorescent switches and logic gates.


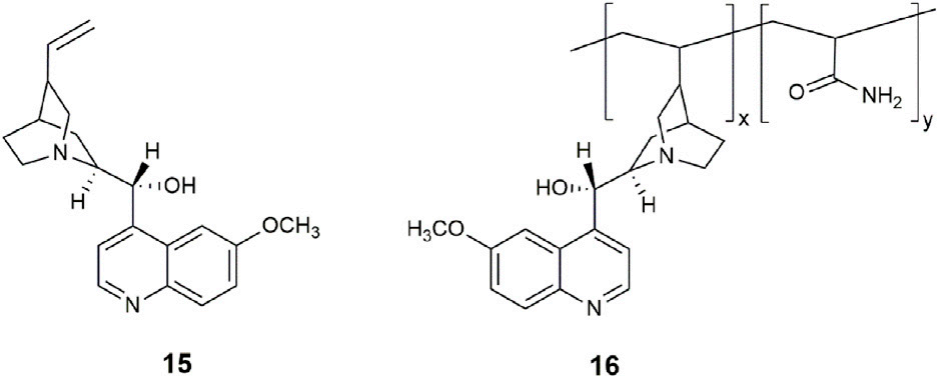


## 3 Challenges and future directions

The detection of three chemical species simultaneously by a single molecule remains a significant challenge. After almost two decades since the first proof-of-concept (Magri et al., 2006a), the number of lab-on-molecules that can detect a congregation of analytes is only a handful. A common rhetoric in the introduction of many sources of literature is that the synthesis of large molecules with one binding site per analyte is too laborious, expensive, and time-consuming. So why are many researchers not so interested in creating sophisticated functional molecules that can detect for three (or more) analytes? Well, of course, synthesis also requires some skill. Organic chemistry remains central to the enterprise of designing, synthesizing, and characterizing mostly carbon-based molecules (Callan et al., 2005). As stated, our observation is that organic chemists as a community, those at the higher end of molecular synthesis, have largely remained aloof from luminescent sensors and switches. An appeal of this Frontiers review, as done two decades ago, is to request this skill base, the creative hard-core organic chemists, to consider contributing to the challenges of luminescent sensors, molecular logic gates, and lab-on-a-molecule development.

As highlighted throughout this review, the majority of lab-on-a-molecule and multi-analyte sensing systems to date have tended to primarily detect for cations, which is understandable within the context of the historic progression of supramolecular and guest–host chemistry. A significant step forward would be for the lab-on-a-molecule that can detect variable combinations of cations, anions, and neutral molecules, and do so ideally in water, and in a competitive media environment.

Here are some ideas for developing the next generation of lab-on-a-molecule systems:

1. More anion receptors have to be made readily available. Numerous cation receptors, such as benzocrown ethers, cyclams, lariat ethers, and cryptands, are commercially available. A successful approach has been to exploit readily available multi-protonable receptors, such as cryptands (Kataev, 2003), or polyamines (Czarnik, 1994) as receptors for inorganic anions and nucleotides.
2. Known ditopic fluorescent logic gates (de Silva et al., 2003; Koskela et al., 2005; Shin et al., 2017) in the scientific literature provide a template for the extension of known logic gates into a lab-on-a-molecule by sequentially adding a third receptor (Magri and de Silva, 2010). After all, the first two-input AND logic gates (de Silva et al., 1993; de Silva et al., 1997b) were an amalgamation of pH (de Silva and Rupasinghe, 1985) and alkaline metal (de Silva and de Silva, 1986) fluorescent indicators (YES logic gates).
3. The exploitation and adoption of one-pot and multicomponent synthesis strategies (Brandner and Müller, 2023) could improve the efficiency and diversity of lab-on-a-molecules.
4. Fluorescent natural products (Duval and Duplais, 2017; Sung and Seok Lee, 2023) can become a source of inspiration as scaffolds for lab-on-a-molecules as has been the case with medicines for well over a century.
